# Knowledge and attitudes towards medicinal cannabis and complementary and integrative medicine (CIM): a survey of healthcare professionals working in a cancer hospital in Australia

**DOI:** 10.1007/s00520-023-08080-z

**Published:** 2023-10-11

**Authors:** Suzanne J. Grant, Maria Gonzalez, Gillian Heller, Sarah Soliman, Gretel Spiegel, Judith Lacey

**Affiliations:** 1 Supportive Care and Integrative Oncology Department, Chris O’Brien Lifehouse Comprehensive Cancer Centre, Missenden Road, Camperdown, Sydney, NSW Australia; 2 NICM Health Research Institute, Western Sydney University, Sydney, NSW Australia; 3 NHMRC Clinical Trials Centre, University of Sydney, Sydney, NSW Australia; 4 School of Science, Western Sydney University, Sydney, NSW Australia; 5 School of Medicine, University of Sydney, Sydney, NSW Australia

**Keywords:** Cannabis, Integrative medicine, Complementary therapies, Cancer, Knowledge, Attitudes

## Abstract

**Purpose:**

We investigated attitudes and practices of healthcare professionals (HCPs) to medicinal cannabis (MC) and complementary and integrative medicine (CIM), including individual therapies, such as acupuncture, massage, herbs, dietary supplements, nutrition and exercise. We explored whether healthcare occupation influenced attitudes to CIM and MC; referral pathways for advice on CIM; and interest in a pharmacy service to evaluate herbs and supplements.

**Methods:**

Cross-sectional survey. All clinical staff at a comprehensive cancer hospital were invited to complete an anonymous questionnaire about CIM and MC. We used descriptive analysis to describe the respondent’s knowledge and attitudes, and Fisher’s exact test to test for differences by occupation, length of time at the hospital and age.

**Results:**

Most of the 116 HCPs respondents supported integrating CIM into cancer care (94.8%) and wanted to learn more (90%) and to understand benefits and contraindications. Most respondents believed that CIM (87.9%) could benefit patients with cancer, and MC could benefit those with advanced cancer (49–51%). Whilst just over half (52.6%) felt confident discussing CIM with patients, only 10% felt they had sufficient knowledge to discuss MC. Most felt they did not have sufficient knowledge to specifically discuss mind and body practices (63.8%) or herbs and supplements (79%). HCPs (63%) would be more inclined to allow use of herbs and supplements with cancer treatment if a pharmacy service was available to evaluate interactions. Occupation, length of time at hospital and age influenced confidence and knowledge about CIM.

**Conclusions:**

The integration of evidence-based CIM and MC into cancer care is hampered by a lack of knowledge of benefits and contraindications, and gaps in education. Effective and safe integration may require targeted development of services such as pharmacy to evaluate the safety of herbs and supplements, and inclusion of cancer specialists who have received training in individual CIM therapies and MC.

**Supplementary Information:**

The online version contains supplementary material available at 10.1007/s00520-023-08080-z.

##  Introduction

Awareness of complementary and integrative medicine (CIM) and the prescription of medicinal cannabis (MC) amongst healthcare professionals (HCPs) is important for safe and effective clinical care of people affected by cancer. An average of 56% of Australians with cancer use CIM, including medicinal cannabis and traditional indigenous and complementary medicines [1, 2]. People with cancer want their cancer care team to be able to discuss CIM and MC; addressing these needs increases satisfaction, confidence and trust in treatment and engagement in their cancer treatment [3–6]. HCPs can be credible sources to provide accurate and trusted information, and their beliefs or biases play an important role in patients’ decisions to share their CIM usage [7–9]. However, recent research reveals that most nurses and oncologists have insufficient knowledge about CIM, leading to variable responses such as discouraging use, or being supportive but without adequate knowledge to refer or prescribe [1, 10]. Little is known about the attitudes of cancer care professionals to different types of CIM therapies or MC.

Medicinal cannabis has been available by medical prescription through a special access or authorised prescriber program in Australia since 2016, with many people with cancer reporting improvement in a range of physical and psychological symptoms [11]. Prescription of MC in Australia is only through medical practitioners, although nurses, psychologists and other healthcare professionals may impact patient's access given their direct involvement in patient care. Whilst prescribing of MC is relatively new in Australia, traditionally consumers were accessing cannabis products without prescription. Reluctance to seek prescribed medicinal cannabis was due to cost, disinterest from the medical profession and stigma regarding cannabis use, with frustration around misinformation leading to non-disclosure [12, 13].

Knowledge and attitudes of health professionals working in cancer care in Australia towards CIM and MC have been examined in several discrete surveys to date. One survey included only pharmacists and their attitude to biologically based complementary therapies in people with cancer [14]. Another study included all healthcare professionals and their attitude only to MC use in cancer[15]. A more recent survey examined attitudes towards CIM as a single group of therapies amongst diverse healthcare professionals working in cancer care [16]. All surveys identified an interest in wanting to learn more about MC or CIM. However, none of these surveys included both CIM and MC, or investigated attitudes towards specific CIM therapies, such as acupuncture, massage, herbs and dietary supplements and exercise therapy. We hypothesised that attitudes and knowledge may differ across discrete CIM therapies.

Our study sought to investigate attitudes and practices of healthcare professionals to the use of different complementary therapies, exercise, nutrition and medicinal cannabis, to understand knowledge gaps, and identify which areas participants were interested in learning about further. We were also interested in whether healthcare occupation influenced attitudes to CIM and MC, referral pathways for advice on CIM and interest in a pharmacy service to evaluate potential interaction between herbs and supplements with cancer treatments. The study was conducted at a hospital that provides a range of CIM as part of a comprehensive integrative oncology service alongside conventional cancer care. To improve service delivery and integration within the hospital, we also investigated the awareness of HCPs of the CIM offerings within the hospital setting.

## Method

This cross-sectional survey investigated attitudes and practices of healthcare professionals, working at a large cancer hospital in Australia, towards complementary and integrative medicine (CIM), exercise and lifestyle medicine and medicinal cannabis (MC). The study received ethics approval from the Sydney Local Area Health District Ethics Committee in May 2019 (HREC/18/RPAH/519). Results are reported according to the STROBE guidelines [17].

### Participants

All eligible (*n*=488) healthcare professional staff working in clinical roles at Chris O’Brien Lifehouse were invited to complete the survey. The total sample number included employees unlikely to respond who were on leave, and casual employees who were not active. The Chris O’Brien Lifehouse in Sydney, Australia is a non-for-profit cancer hospital and services over 15,000 patients per year, and has a dedicated integrative oncology service [18].

### Survey design

A questionnaire was developed by four of the authors (SG, SS, JL and MG) based on a literature review. The questionnaire comprised 26 questions with four sections: demographics (5 items), knowledge and attitudes to CIM (9 items) and medicinal cannabis (7 items), and knowledge of integrative oncology services within the hospital (6 items) (Online Resource 1). Questions were adapted from the validated Complementary and Integrative Health Assessment for Practitioners (CIHAP) which assesses HCPs current knowledge of CM and their interest in integrating CM into their practices [19]. Other questions were adapted from a survey used to understand oncologists’ practices around CM [20]. Additional questions were included about medicinal cannabis; these questions were adapted from other surveys [21–23].

The survey was pre-tested with five healthcare professionals considered representative of the respondents, reviewed and tested again in a different group prior to distribution. These healthcare professionals did not complete the final survey.

Complementary therapies are defined as a group of diverse medical and healthcare interventions, practices, products or disciplines that are not generally part of conventional medicine. This includes natural products (such as herbs, vitamins and minerals) and mind and body practices (yoga, mindfulness, massage, acupuncture, reflexology qi gong, tai chi). Integrative oncology was defined as a patient-centred, evidence-informed field of comprehensive cancer care that uses mind-body practices, natural products and lifestyle modifications from different traditions alongside conventional cancer treatments [24].

### Procedure

Participation was voluntary. HCPs were invited to complete the self-administered, anonymous survey via the sharing of a link and QR code through staff email circulars, distribution of flyers throughout staff areas of the hospital and verbal communications about the survey at staff meetings. The invitation link was available between 1 May and 30 August 2022. Respondents were asked to complete the survey only once, but multiple participation was not able to be prevented as to do so would have violated the anonymous condition of the survey. No cookies were collected, and no data was collected that would enable the identification of individuals. Qualtrics (Qualtrics, Provo, UT) was used to administer the survey. A consent button, included at the start of the survey, informed participants about the survey and requested their consent to continue. Estimated completion time for the survey was 8–10 min.

### Statistical analysis

Answers to questions were recorded in Qualtrics, exported as a .csv file and analysed using the statistical programming language R*.* Answers based on the modified Likert scales were collapsed into dichotomous categories of agree and disagree and percentages calculated for each. We tested whether agreement with statements was related to gender (male vs female), age (up to 50 years vs 51 years or more) or occupation using simple binomial regression for crude odds ratios (ORs) and multiple binomial regression for ORs adjusted for all other variables.

## Results

Of the 488 eligible healthcare professionals working within the hospital, 116 responded to the survey and provided demographic data (Table 1). The majority of participants were female (76%) and in the 31–50 year old age group (48%).

**Table 1:** Demographic characteristics of respondents

	*n* = 116
Age	
<31	29 (33%)
31–50	43 (48%)
>51	17 (19%)
Prefer not to say	27
Gender	
Female	68 (76%)
Male	22 (24%)
Prefer not to say	26
Occupation	
Nurse	53 (46%)
Allied health professional	20 (17%)
Oncologist	16 (14%)
Pharmacist	11 (9.5%)
Other	9 (7.8%)
Supportive care	4 (3.4%)
Surgeon	3 (2.6%)
Length of time at hospital	
More than 12 months	70 (75%)
Less than 12 months	23 (25%)
Unknown	23
Place of work	*n* = 149^1^
Inpatient wards	40 (31%)
Day therapy	24 (18%)
Outpatient clinics	21 (16%)
Radiation oncology	18 (14%)
Pharmacy	11 (8.4%)
Clinical trials	5 (3.8%)
Surgical theatres	8 (6.1%)
Living Room	4 (3.1%)

^1^Participants could select more than one place of work

Healthcare professional participants included 53 nurses, 16 oncologists (including radiation and medical oncology), 11 pharmacists, 20 allied health professionals (including dietitians, exercise physiologists, physiotherapists and psychologists) and 16 other healthcare professionals (including surgeons, palliative and supportive care staff). Participants worked primarily in the hospital’s inpatient wards (31%) and the day therapy (chemotherapy) suites (18%), radiation oncology and outpatient clinics.

###  Knowledge and attitudes to CIM and medicinal cannabis

Nearly all respondents were supportive of the integration of complementary therapies into cancer care (94.8%) and agreed that these therapies can be beneficial to patients with cancer (87.9%) (Table 2). Respondents perceived CIM therapies to have benefit for depression, anxiety and stress management (97.4%). Whilst just over half (52.6%) felt confident discussing complementary therapies with patients, the remainder (48%) were undecided or not confident. Most HCPs felt that they did not have sufficient knowledge to discuss mind and body practices (63.8%) or herbs and supplements (79%) but wanted to learn more about complementary therapies (89.7%).

**Table 2:** Knowledge and attitudes to complementary therapies and MC in cancer care^*^

	All respondents *n*=116 (%)	Allied health *n*=20	Nurse *n*=53	Oncologist *n*=16	Pharmacist *n*=11
	Agree *n*(%)				
1. I am supportive of the integration of complementary therapies into a cancer setting	110 (94.8)	20 (100)	52 (98)	15 (94)	10 (91)
2. I am confident discussing complementary therapies with patients	61 (52.6)	12 (60)	31 (58)	5 (31)	5 (45)
3. Many complementary therapies (for example, massage, yoga, acupuncture and mindfulness) have beneficial effects on psychological symptoms such as depression and anxiety and stress management	113 (97.4)	20 (100)	52 (98)	16 (100)	11 (100)
4. I feel I have sufficient knowledge about mind and body practices such as yoga, mindfulness and therapies such as massage, reflexology and acupuncture to advise patients on benefits and contraindications	42 (36.2)	10 (50)	17 (32)	3 (19)	3 (27)
5. I feel I have sufficient knowledge about herbs and supplements to advise patients on benefits and contraindications	24 (20.7)	5 (25)	7 (13)	3 (19)	6 (55)
6. I believe complementary therapies can be beneficial to patients with cancer	102 (88)	20 (100)	48 (91)	14 (88)	8 (73)
7. I want to learn more about complementary therapies in cancer care	104 (90)	20 (100)	51 (96)	13 (81)	10 (91)
8. I have sufficient knowledge about medicinal use of cannabis to make recommendations to oncology patients	12 (10.3)	4 (24)	2 (5)	1 (8)	4 (40)
9. Healthcare professionals should receive continuing professional development about medicinal cannabis	88 (93)	17 (100)	42 (98)	9 (69)	10 (100)
10. There is sufficient scientific evidence supporting the efficacy of medicinal cannabis	45 (47)	7 (41)	26 (60)	8 (62)	5(50)
11. My attitude towards prescribing medical cannabis has changed	31 (33)	6 (35)	16 (37)	3 (23)	3 (30)
12. I am familiar with the endocannabinoid system	17 (18)	2 (12)	3 (7)	5 (38)	3 (30)
In your opinion or according to your experience, which of these cancer patient populations can benefit from medicinal cannabis:					
13. Patients with advanced disease receiving supportive care alone/end-of-life care	55 (50.9)	14 (70)	24 (45)	10 (62)	3 (27)
14. Patients receiving active disease-modifying treatment for advanced/metastatic cancer with refractory symptoms	57 (49.1)	12 (60)	23 (43)	11 (69)	4 (36)
15. Cancer survivors with persisting refractory (difficult to manage) symptoms	51 (43.9)	11 (55)	21 (40)	6 (38)	6 (55)
16.Early-stage patients with treatment-related refractory side effects or symptoms	45 (38.7)	9 (45)	22 (42)	7 (44)	3 (27)
17. Any patient with a cancer diagnosis (independent of symptom burden)	26 (22.4)	3 (15)	17 (32)	1 (6)	3 (27)
18. I do not know/cannot answer	15 (12.9)	3 (17)	9 (17)	1 (6)	1 (9)

*Provides the numbers who ‘Agreed’ or ‘Strongly Agreed’ with the statements; total respondents *n*=116; occupation groups >10 respondents were included as distinct categories

More than half of the respondents agreed that there was benefit from the use of medicinal cannabis in those with advanced cancer (59/116), including those receiving active treatment (57/116) (Table 2). Slightly less than half of respondents thought cancer survivors with refractory symptoms could benefit (51/116).

For the statements with substantial percentage differences amongst professions (2, 4 and 5) Fisher’s Exact test was carried out to test for differences by occupation, length of time at the hospital and age. For Statements 2 and 4 there was no significant difference amongst occupations. For Statement 5 (*I feel I have sufficient knowledge about herbs and supplements to advise patients on benefits and contraindications*) a larger percentage of pharmacists (*p*=0.008) compared to the other professions felt they had sufficient knowledge about herbs and supplements to advise patients on benefits and contraindications. Those working in the hospital for more than 12 months were more likely to agree with Statements 2 (*p*<0.001), 4 (*p*=0.038) and 5 (*p*=0.03). Older participants were also more likely to agree with Statements 2 (*p*=0.005), Statement 4 (*p*=0.006) and Statement 5 (*p*=0.024).

In the overall sample, the majority of participants wanted to learn more about each of the therapies included in the survey (Table 3). Whilst knowledge on all types of CIM therapies desired, herbs (94/116) and dietary supplements (94/116) had slightly higher interest than other therapies. Few participants indicated already had enough knowledge or were (6/113) not being interested (3/116) in learning any further about CIM and lifestyle interventions.

**Table 3:** Desire to learn more about CIM amongst participants

I want to learn more about the benefits and contraindications for cancer patients of:	All respondents *n* = 116 (%)	Allied health *n*=20 (%)	Nurse *n*=53 (%)	Oncologist *n*=16 (%)	Pharmacist *n*=11 (%)
Dietary supplements	94 (81)	15 (75)	46 (87)	12 (75)	10 (91)
Herbs	94 (81)	16 (80)	47 (89)	10 (62)	9 (82)
Mind body therapies	89 (77)	16 (80)	47 (89)	10 (62)	8 (73)
Nutrition	87 (75)	14 (70)	44 (83)	10 (62)	8 (73)
Acupuncture	86 (74)	15 (75)	47 (89)	11 (69)	6 (55)
Exercise	80 (69)	13 (65)	43 (81)	8 (50)	7 (64)
Massage and reflexology	80 (69)	15 (75)	45 (85)	6 (38)	6 (55)
Have enough knowledge about complementary therapies and lifestyle interventions	6 (5)	0 (0)	1 (2)	1 (6)	0 (0)
Not interested in learning any further about complementary therapies and lifestyle interventions	3 (3)	1 (5%)	1 (2%)	2 (12%)	0 (0)
I have seen patients improve faster when they used a complementary therapy along with conventional health practices	39 (36)	8 (42)	22 (44)	2 (13)	3 (27)
I feel it is essential to network and build relationships with complementary therapies, exercise oncology and integrative oncology providers within the hospital	95 (89)	18 (95)	47 (94)	11 (73)	10 (91)
I feel my professional training has prepared me for integration of complementary therapies and lifestyle medicine	36 (34)	10 (53)	17 (34)	1 (6.7)	4 (36)

Table 3 reports attitudes towards CIM and lifestyle medicine amongst HCPs. The majority (60.7%) of participants were undecided about whether they had seen patients improve faster when using CIM along with conventional health practices. Nearly all (89%) participants felt it was essential to network and build relationships with providers within the hospital. A third (33.6%) of participants agreed with feeling that their professional training had prepared them for integration of CIM and lifestyle medicine into their practice.

We sought to determine whether any significant differences existed for each of these statements based on occupation, length of time working at the hospital and age. A minimal difference was found for occupation (*p*=0.045), whilst no effect was found for length of time at the hospital or age.

###  Practice—CIM and MC recommendations, prescribing and referrals

A total of 85 HCPs (75%) used CIM and lifestyle therapies to support their own health (Table 4), with 44% using massage/reflexology, nutrition (43%), exercise (64%) and dietary supplements (33%). CIM use was lowest amongst oncologists, although there was no statistical association between occupation and CIM use (Fisher’s exact test *p*=0.196). Few respondents reported using acupuncture (16%) or herbs (16%). Most HCPs (65%) would not advise against any of the individual CIM or against MC. Herbs (14%) and dietary supplements (7%) were the CIM therapies most likely to be advised against. Amongst the different occupations, more oncologists compared to the other occupations recommended against herbs (40%) and dietary supplements (27%), though numbers are small overall. There was no association found between personal use of CIM and advice against CIM or MC use to cancer patients.

**Table 4:** Use of CIM, lifestyle and diet amongst HCPs, recommendations for and against

	All respondents *n*=116 (%)	Nurses *n*=53 (%)	Allied health *n*=20 (%)	Oncologists *n*=16 (%)	Pharmacists *n*=11 (%)
Do you use complementary therapies, lifestyle and diet based therapies to support your own health?	85 (75)	40 (75)	18 (90)	9 (56)	7 (64)
Acupuncture	15 (13)	4 (8)	4 (20)	1 (6)	1 (9)
Dietary supplements	38 (33)	16 (30)	8 (40)	3 (19)	5 (45%)
Exercise	74 (64)	16 (80)	35 (66)	8 (50)	6 (55)
Herbs	19 (16)	8 (15)	3 (15)	1 (6)	4 (36)
Massage and reflexology	51 (44)	24 (45)	15 (75)	1 (6)	4 (36)
Mind body therapies	43 (37)	23 (43)	9 (45)	2 (12)	5 (45)
Nutrition	50 (43)	24 (45)	10 (50)	5 (31)	4 (36)
None of the above	29 (25)	13 (25)	2 (10)	7 (44)	4 (36)
Which, if any, complementary therapies or lifestyle changes would you strongly advise against patient use?					
Mind body therapies	2 (2)	0 (0)	(0)	1 (6)	0 (0)
Herbs	16 (14)	4 (8)	2 (10)	6 (38)	2 (18)
Dietary supplements	8 (7)	2 (4)	0 (0)	4 (25)	1 (9)
Massage and reflexology	1 (1)	0 (0)	0 (0)	1 (6)	0 (0)
Acupuncture	3 (3)	2 (4)	0 (0)	0 (0)	1 (9.1)
Exercise	1 (1)	0 (0)	0 (0)	1 (6)	0 (0)
Nutrition	2 (2)	0 (0)	0 (0)	2 (2)	0 (0)
Medicinal cannabis	3 (3)	0 (0)	0 (0)	2 (12)	0 (0)
None of the above	75 (65)	40 (75)	15 (75)	7 (44)	8 (73)

Participants were asked who they recommended their patients seek advice about CIM from (Table 5). The highest rated was an integrative and supportive care medical specialist (31%), followed by an oncologist (18%). The majority of participants (63% yes definitely, 23% yes slightly) indicated that they would be more inclined to recommend or allow the use of some CIM if a pharmacy service existed which evaluated the potential interaction between herbs and supplements with cancer treatments.

**Table 5:** Seeking advice about CIM

Who do you recommend your patients seek advice about CIM from?	Yes *n*=294* (%)
Integrative and supportive care medical specialist	82 (31)
Oncologist	49 (18)
Dietitian	40 (15)
Pharmacist	29 (11)
GP	28 (11)
Clinical nurse consultant	27 (10)
No one. I discuss this with them	11 (4.1)
If there was a pharmacy service to evaluate the potential interaction between herbs and/or supplements with cancer treatments, would this make you more inclined to recommend or allow use of some of these therapies	*n* = 116
I do not know	6 (5.6)
Will not affect my decision	9 (8.4)
Yes definitely	67 (63)
Yes slightly	25 (23)
Unknown	9

*More than one response permitted

A large percentage of HCPs (71%) indicated knowledge of their patients being prescribed medicinal cannabis (Online Resource 2). When asked about knowledge regarding self-prescribing of medicinal cannabis, slightly less than half (41%) were aware that their patients had been self-prescribing cannabis and a similar percentage did not know (46%). A small percentage (6.3%) were not aware whether their patients were self-prescribing cannabis. Patient experiences and medical literature were most influential in changing attitudes towards medicinal cannabis (Online Resource 2).

Participants were asked whether they agreed that they had sufficient knowledge to refer patients to the Integrative Oncology and Supportive Care Department at Chris O’Brien Lifehouse (Online Resource 2). Between approximately a third and two-thirds of participants agreed that they had sufficient knowledge to refer patients to the service. Knowledge for referral was lowest for exercise physiology (34%) and survivorship program (38%). The level of agreement was similar between different occupations except for pharmacists, who tended to agree less than those in other occupations.

###  Symptom management with medicinal cannabis

Over half of the participants felt that medicinal cannabis may be helpful for all the cancer-related symptoms included in the survey (Online Resource 2). Cancer and treatment-related nausea and vomiting were the most common symptom; HCPS agreed MC may be beneficial, 84.2% selecting cancer-related nausea and 83.2% selecting chemo-related nausea/vomiting.

**Fig. 1:**
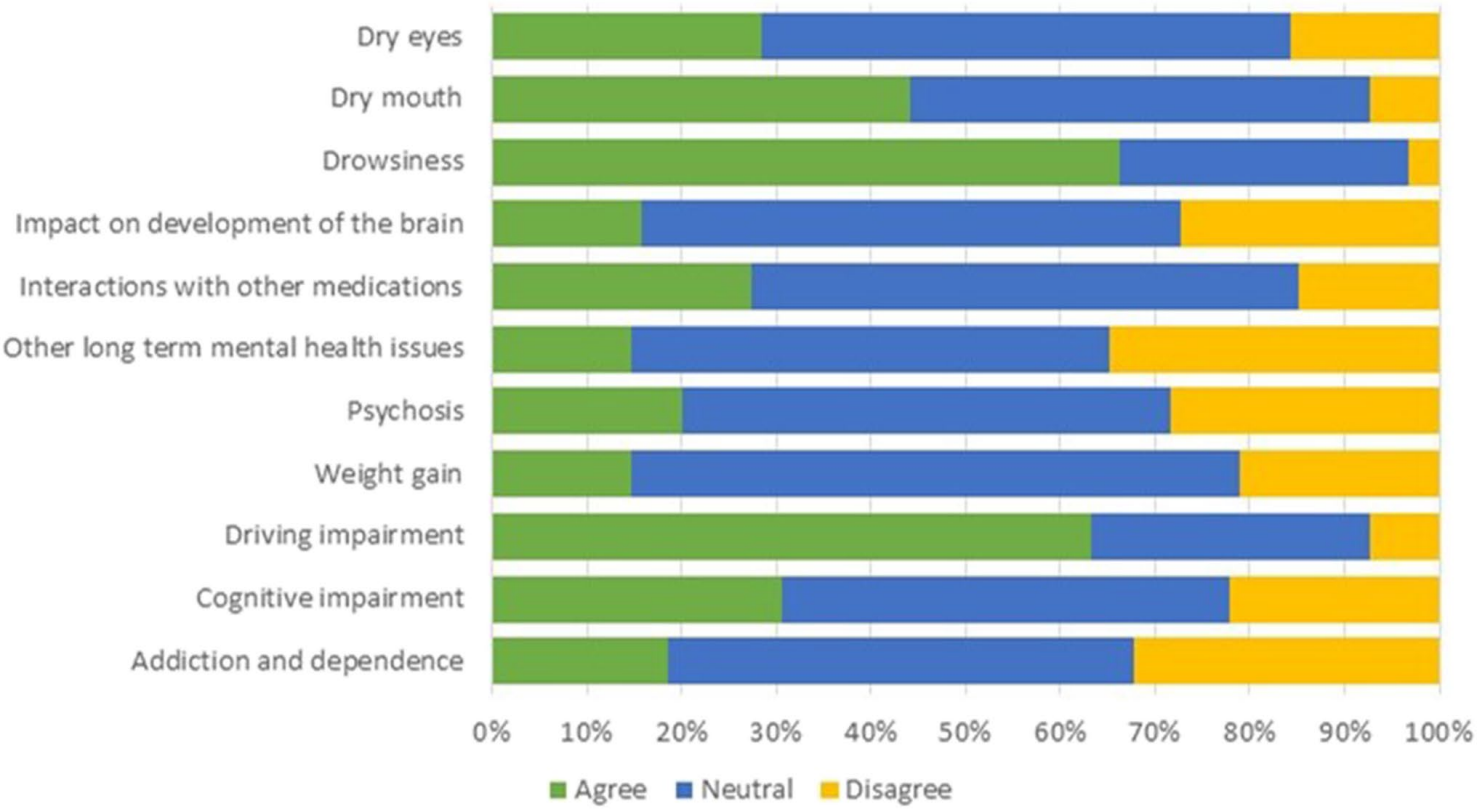
Agreement of HCPs with the major side effects of medicinal cannabis

The majority of participants identified driving impairment (63.2%) and drowsiness (66.3%) as a major side effect of MC. For most side effects, healthcare professionals neither agreed nor disagreed (Fig. 1).

## Discussion

Our study found that nearly all 116 respondents supported the integration of complementary and integrative medicine (CIM) into cancer care, and believed these therapies can be beneficial to patients with cancer, but the majority of respondents did not feel they had adequate knowledge to advise patients on CIM. For medicinal cannabis (MC), attitudes were more ambiguous, with only half of respondents agreeing that there was adequate evidence for the efficacy of MC, or that MC was beneficial to people with cancer. Nearly all respondents wanted to learn more about complementary therapies; this is consistent with other studies [25]. Only 33% of healthcare professionals felt prepared to integrate CIM into their work. As hypothesised, HCPs knowledge of individual CIM therapies varied between mind-body, and herbs and supplements.

The majority of respondents agreed CIM has beneficial effects for people with cancer. Nearly all respondents believed CIM was beneficial to people with cancer, and mind-body therapies had beneficial effects on psychological symptoms such as depression and anxiety and stress management. MC may benefit cancer-related symptoms, and this was higher for symptoms such as managing cancer and treatment-related nausea and appetite. Side effects of MC were thought to be primarily driving impairment and drowsiness but there was uncertainty around other impacts.

With only a few exceptions, knowledge and attitudes to CIM and MC in our study were not influenced by age, gender, occupation or length of time at the hospital. Those respondents who had worked at the hospital longer and were older were more likely to report confidence in discussing CIM with patients and reported having sufficient knowledge about mind-body practices and herbs and supplements.

Knowledge gaps were highest for MC, with only 10% reporting they had adequate knowledge to recommend or 17% were familiar with the endocannabinoid system. Only 20% of HCPs were confident advising on the benefits and contraindications of herbs and supplements; confidence was slightly higher for mind-body practices (36%). All HCPs wanted to learn more about the benefits of CIM and MC. Interest was highest for dietary supplements (81%) and herbs (81%), with the majority of oncologists wanting to learn about the benefits and contraindications of dietary supplements and herbs.

Despite limited confidence in benefits and contraindications of herbs and supplements, only 7–14% of the HCPs would advise against the use of herbs and dietary supplements. In a pooled prevalence of studies of people with cancer, 22% used herbal medicine, and this is higher in certain population groups such as women with breast cancer where 41% reported use of herbal medicine [26, 27]. Dietary supplement use is higher, with studies indicating use by almost one in two people with cancer (including those receiving cancer treatment), with a slightly lower but significant (36%) level of consumption in men [28–30]. Despite this prevalence of use in Australian cancer patients, respondents in our study did not feel they were equipped to advise on herbs and supplements, and this area received the highest interest by HCPs in learning more, compared to mind-body therapies.

Advice regarding the safe use of herbal medicine or dietary supplement use during cancer treatment is an important part of comprehensive cancer care. Yet only 16 cancer services in Australia have dedicated healthcare practitioners providing advice on the use of any CIM [31]. In our study, there was strong support for a pharmacy service to advise on the potential interaction and safety of herbs and/or supplements with cancer treatments. The majority of respondents stated this would make them more likely to recommend or allow use. More than half of the pharmacists in our survey reported that they were confident in advising on herbs and supplements. For CIM in general, respondents in our study were most likely to refer patients to the integrative and supportive care specialist within the hospital. Taken together, these findings indicate that the safety and choices of people with cancer and their supportive care can be greatly enhanced by providing a level of CIM and MC specialist knowledge within a comprehensive cancer setting.

Meeting cancer patient expectations, cultural preferences, beliefs and information needs improves patient outcomes [32]. One in two Australians with cancer uses CIM; it aligns with their personal values, beliefs and cultural identity [33]. People use CIM to help cope with the side effects of conventional cancer treatments, improve survival and long-term outcomes and support their mental health, wellbeing, weight management, self-efficacy and quality of life throughout the cancer continuum [7, 34–36]. Additionally, patients receiving treatment at an institution that supports an IO program may have improved survival [33, 36–38]. Our survey, in line with other studies, shows that the education, integration, pathways and translation of evidence into practice are major barriers to preferences being expressed by HCPs and people with cancer[16].

The establishment of designated cancer treatment centres in Australia with links to rural and remote centres may provide the basis for speciality advice on integrative oncology to patients and practitioners. Similar to the program of the National Cancer Institute designated cancer centres in the US have developed or are developing integrative oncology programs to assist, along with guidelines to support providers who participate in these programs [39, 40]. This would require the training of healthcare professionals in integrative oncology; competencies and training options are emerging [41, 42].

Whilst knowledge and attitudes of general practitioners to MC have been assessed [43], no studies conducted in Australia have explored attitudes of oncology HCPs. The findings in our study were similar to other studies conducted in Europe, where oncology healthcare professionals increasingly agree that MC reduces patient suffering and has benefits, particularly in people with advanced cancer [23].

The study was conducted at a hospital that has provided a range of CIM alongside conventional cancer care since it opened in 2013 and more recently prescription of MC. Through this exposure, we anticipated that the HCPs surveyed may have different knowledge and attitudes towards CIM compared to participants in other surveys. In other surveys 58–90% of HCPs reported having inadequate knowledge to answer questions about CIM, compared to 51% of HCPs in our study reporting that they felt confident in discussing CIM with patients[16]. However, this knowledge did not extend to side effects of MC or the endocannabinoid system, and most respondents did not have sufficient knowledge to make recommendations about MC use to people with cancer.

## Limitations

Our study had several limitations. We used convenience sampling from a single institution, and this may impact external validity. Participation in the survey was voluntary. However, the sample may not have been representative of the hospital population resulting in selection bias. The survey was administered in an anonymous and confidential manner which may mitigate some bias. There are no validated tools for measuring attitudes and beliefs to the use of cannabis in cancer care which may have resulted in information bias.

## Conclusion

The uptake and integration of evidence-based and informed CIM and MC by oncologists and other HCPs in cancer care are hampered by a lack of knowledge of benefits and contraindications, gaps in education and training and the lack of adequate referral pathways [44]. The results of this survey will inform the development of ongoing education activities, knowledge sharing and research activities. Effective and safe integration of CIM and MC may require the targeted development of services such as pharmacy to evaluate the safety of herbs and supplements with a focus on drug-herb interactions and inclusion of cancer specialists who have received specific training in CIM and MC [45]. The targeted development of pharmacy and training of dedicated HCPs to provide advice on CIM and MC would support informing the choice of 1 in 2 Australians with cancer who use CIM.

###  Supplementary information

ESM 1

ESM 2

ESM 3

## Data Availability

Data is available on special request.
