# Supplementary material for: Knowledge and attitudes towards medicinal cannabis and complementary and integrative medicine (CIM): a survey of healthcare professionals working in a cancer hospital in Australia
Source: Support Care Cancer. 2023 Oct 11;31(11):623. doi: 10.1007/s00520-023-08080-z (PMC10567955; doi:10.1007/s00520-023-08080-z)
Supplement: Supplementary file 1 — ESM 1 [file 520_2023_8080_MOESM1_ESM.docx]

**Knowledge and attitudes toward medicinal cannabis and complementary and integrative medicine (CIM): a survey of healthcare professionals working in a cancer hospital in Australia**

Suzanne J GRANT^1,2^, Maria GONZALEZ^1, 2^, Gillian HELLER^3^, Sarah SOLIMAN^4^, Gretel SPIEGEL^1^, Judith LACEY^1,2,5^

1. Supportive Care and Integrative Oncology Department, Chris O’Brien Lifehouse Comprehensive Cancer Centre, Sydney, NSW, Australia
2. NICM Health Research Institute, Western Sydney University, Sydney, NSW, Australia
3. NHMRC Clinical Trials Centre, University of Sydney, NSW, Australia
4. School of Science, Western Sydney University, NSW, Sydney, Australia
5. University of Sydney, School of Medicine, NSW, Australia

**Corresponding author:**

Suzanne Grant, Chris O’Brien Lifehouse

Missenden Road, Camperdown NSW, Australia

e. [s.grant@westernsydney.edu.au](mailto:s.grant@westernsydney.edu.au)

**ABSTRACT**

**Purpose:** We investigated attitudes and practices of health care professionals (HCPs) to medicinal cannabis (MC) and complementary and integrative medicine (CIM), including individual therapies, such as acupuncture, massage, herbs, dietary supplements, nutrition, and exercise. We explored whether healthcare occupation influenced attitudes to CIM and MC; referral pathways for advice on CIM; and interest in a pharmacy service to evaluate herbs and supplements.

**Methods:** Cross-sectional survey. All clinical staff at a comprehensive cancer hospital were invited to complete an anonymous questionnaire about CIM and MC. We used descriptive analysis to describe the respondent’s knowledge and attitudes, and Fisher’s Exact test to test for differences by occupation, length of time at the hospital and age.

**Results:** Most of the 116 HCPs respondents supported integrating CIM into cancer care (94.8%), wanted to learn more (90%) and to understand benefits and contraindications. Most respondents believed that CIM (87.9%) could benefit patients with cancer, and MC could benefit those with advanced cancer (49%-51%). While just over half (52.6%) felt confident discussing CIM with patients, only 10% felt they had sufficient knowledge to discuss MC. Most felt they did not have sufficient knowledge to specifically discuss mind and body practices (63.8%) or herbs and supplements (79%). HCPs (63%) would be more inclined to allow use of herbs and supplements with cancer treatment if a pharmacy service was available to evaluate interactions. Occupation, length of time at hospital and age influenced confidence and knowledge about CIM.

**Conclusions:** The integration of evidence based CIM and MC into cancer care is hampered by a lack of knowledge of benefits and contraindications, and gaps in education. Effective and safe integration may require targeted development of services such as pharmacy to evaluate the safety of herbs and supplements, and inclusion of cancer specialists who have received training in individual CIM therapies and MC.

**Key words:** cannabis, integrative medicine, complementary therapies, cancer, knowledge, attitudes

**Introduction**

Awareness of complementary and integrative medicine (CIM) and the prescription of medicinal cannabis (MC) among health care professionals (HCPs) is important for safe and effective clinical care of people affected by cancer. An average of 56% of Australians with cancer use CIM, including medicinal cannabis and traditional indigenous and complementary medicines [22, 29]. People with cancer want their cancer care team to be able to discuss CIM and MC, addressing these needs increases satisfaction, confidence and trust in treatment and engagement in their cancer treatment [3, 30, 32, 35]. HCPs can be credible sources to provide accurate and trusted information, and their beliefs or biases play an important role in patients’ decisions to share their CIM usage [13, 31, 40]. However, recent research reveals that most nurses and oncologists have insufficient knowledge about CIM, leading to variable responses such as discouraging use, or being supportive but without adequate knowledge to refer or prescribe [9, 22]. Little is known about the attitudes of cancer care professionals to different types of CIM therapies or MC.

Medicinal cannabis has been available by medical prescription through a special access or authorised prescriber program in Australia since 2016, with many people with cancer reporting improvement in a range of physical and psychological symptoms [5]. Prescription of MC in Australia is only through medical practitioners, although nurses, psychologists and other healthcare professionals may impact patient's access given their direct involvement in patient care. Whilst prescribing of MC is relatively new in Australia, traditionally consumers were accessing cannabis products without prescription. Reluctance to seek prescribed medicinal cannabis was due to cost, disinterest from the medical profession and stigma regarding cannabis use, with frustration around misinformation leading to non-disclosure [26, 42].

Knowledge and attitudes of health professionals working in cancer care in Australia towards CIM and MC, have been examined in several discrete surveys to date. One survey included only pharmacists and their attitude to biologically based complementary therapies in people with cancer [16]. Another study included all healthcare professionals and their attitude only to MC use in cancer[17]. A more recent survey examined attitudes towards CIM as a single group of therapies among diverse healthcare professionals working in cancer care [24]. All surveys identified an interest in wanting to learn more about MC or CIM. However, none of these surveys included both CIM and MC, or investigated attitudes toward different CIM therapies, such as acupuncture, massage, herbs and dietary supplements and exercise therapy. We hypothesised that attitudes and knowledge may differ across discrete CIM therapies.

Our study sought to investigate attitudes and practices of healthcare professionals to the use of different complementary therapies, exercise, nutrition and medicinal cannabis, to understand knowledge gaps, and identify which areas participants were interested in learning about further. We were also interested in whether healthcare occupation influenced attitudes to CIM and MC, referral pathways for advice on CIM, and interest in a pharmacy service to evaluate potential interaction between herbs and supplements with cancer treatments. The study was conducted at a hospital that has provided a range of CIM as part of a comprehensive integrative oncology service alongside conventional cancer care. To improve service delivery and integration within the hospital, we also investigated the awareness of HCPs of the CIM offerings within the hospital setting.

**Method**

This cross-sectional survey investigated attitudes and practices of healthcare professionals, working at a large cancer hospital in Australia, toward complementary and integrative medicine (CIM), exercise and lifestyle medicine and medicinal cannabis (MC). The study received ethics approval from the Sydney Local Area Health District Ethics Committee in May 2019 (HREC/18/RPAH/519). Results are reported according to the STROBE guidelines [14].

*Participants:* All eligible (n=488) healthcare professional staff working in clinical roles at Chris O’Brien Lifehouse were invited to complete the survey. The total sample number included employees unlikely to respond who were on leave, and casual employees who were not active. The Chris O’Brien Lifehouse in Sydney, Australia is a non-for-profit cancer hospital and services over 15,000 patients per year, and has a dedicated integrative oncology service [15].

*Survey design:* A questionnaire was developed by four of the authors (SG, SS, JL and MG) based on a literature review. The questionnaire comprised 26 questions with four sections: demographics (5 items), knowledge and attitudes to CIM (9 items) and medicinal cannabis (7 items), and knowledge of integrative oncology services within the hospital (6 items) (Online Resource 1). Questions were adapted from the validated Complementary and Integrative Health Assessment for Practitioners (CIHAP) which assesses HCPs current knowledge of CM and their interest in integrating CM into their practices [6]. Other questions were adapted from a survey used to understand oncologists’ practices around CM [7]. Additional questions were included about medicinal cannabis, these questions were adapted from other surveys [2, 8, 12].

The survey was pre-tested with five healthcare professionals considered representative of the respondents; reviewed and tested again in a different group prior to distribution. These healthcare professionals did not complete the final survey.

Complementary therapies are defined as a group of diverse medical and health care interventions, practices, products or disciplines that are not generally part of conventional medicine. This includes natural products (such as herbs, vitamins and minerals) and mind and body practices (yoga, mindfulness, massage, acupuncture, reflexology qi gong, tai chi). Integrative oncology was defined as a patient-centered, evidence-informed field of comprehensive cancer care that uses mind-body practices, natural products, and lifestyle modifications from different traditions alongside conventional cancer treatments [43].

*Procedure:* Participation was voluntary. HCPs were invited to complete the self-administered, anonymous survey via the sharing of a link and QR code through staff email circulars, distribution of flyers throughout staff areas of the hospital and verbal communications about the survey at staff meetings. The invitation link was available between 1 May and 30 August 2022. Respondents were asked to complete the survey only once but multiple participation was not able to be prevented as to do so would have violated the anonymous condition of the survey. No cookies were collected and no data was collected that would enable the identification of individuals. Qualtrics (Qualtrics, Provo, UT) was used to administer the survey. A consent button, included at the start of the survey, informed participants about the survey and requested their consent to continue. Estimated completion time for the survey was 8-10 mins.

*Statistical analysis*

Answers to questions were recorded in Qualtrics, exported as a .csv file and analysed using the statistical programming language R*.* Answers based on the modified Likert scales were collapsed into dichotomous categories of agree and disagree and percentages calculated for each. We tested whether agreement with statements was related to gender (male vs female), age (up to 50 years vs 51 years or more), or occupation using simple binomial regression for crude odds ratios (ORs) and multiple binomial regression for ORs adjusted for all other variables.

**Results**

Of the 488 eligible health care professionals working within the hospital, 116 responded to the survey and provided demographic data (Table 1). The majority of participants were female (76%) and in the 31–50 year old age group (48%).

Health care professional participants included 53 nurses, 16 oncologists (including radiation and medical oncology), 11 pharmacists, 20 allied health professionals (including dietitians, exercise physiologists, physiotherapists and psychologists) and 16 other health care professionals (including surgeons, palliative and supportive care staff). Participants worked primarily in the hospital’s inpatient wards (31%) and the day therapy (chemotherapy) suites (18%), radiation oncology and outpatient clinics.

**Table 1.** Demographic characteristics of respondents

|  | | n = 116 | |
| --- | --- | --- | --- |
| **Age** | |  | |
| <31 | | 29 (33%) | |
| 31–50 | | 43 (48%) | |
| >51 | | 17 (19%) | |
| Prefer not to say | | 27 | |
| **Gender** | |  | |
| Female | | 68 (76%) | |
| Male | | 22 (24%) | |
| Prefer not to say | | 26 | |
| **Occupation** | |  | |
| Nurse | | 53 (46%) | |
| Allied health professional | | 20 (17%) | |
| Oncologist | | 16 (14%) | |
| Pharmacist | | 11 (9.5%) | |
| Other | | 9 (7.8%) | |
| Supportive care | | 4 (3.4%) | |
| Surgeon | | 3 (2.6%) | |
| **Length of time at hospital** | |  | |
| More than 12 months | | 70 (75%) | |
| Less than 12 months | | 23 (25%) | |
| Unknown | | 23 | |
| **Place of work** | | n = 149^1^ | |
| Inpatient wards | | 40 (31%) | |
| Day therapy | | 24 (18%) | |
| Outpatient clinics | | 21 (16%) | |
| Radiation oncology | 18 (14%) | |  |
| Pharmacy | 11 (8.4%) | |  |
| Clinical trials | 5 (3.8%) | |  |
| Surgical theatres | 8 (6.1%) | |  |
| Living Room | 4 (3.1%) | |  |

1. Participants could select more than one place of work.

***Knowledge and attitudes to CIM and Medicinal Cannabis***

Nearly all respondents were supportive of the integration of complementary therapies into cancer care (94.8%) and agreed that these therapies can be beneficial to patients with cancer (87.9%) (Table 2). Respondents perceived CIM therapies to have benefit for depression, anxiety and stress management (97.4%). While just over half (52.6%) felt confident discussing complementary therapies with patients, the remainder (48%) were undecided or not confident. Most HCPs felt that they did not have sufficient knowledge to discuss mind and body practices (63.8%) or herbs and supplements (79%) but wanted to learn more about complementary therapies (89.7%).

More than half of the respondents agreed that there was benefit from the use of medicinal cannabis in those with advanced cancer (59/116), including those receiving active treatment (57/116) (Table 2). Slightly less than half of respondents thought cancer survivors with refractory symptoms could benefit (51/116).

**Table 2.** Knowledge and attitudes to complementary therapies and MC in cancer care^*^

|  | All respondents  n=116 (%) | Allied Health  n=20 | Nurse  n=53 | Oncologist  n=16 | Pharmacist  n=11 |
| --- | --- | --- | --- | --- | --- |
|  | **Agree n(%)** | | | | |
| 1.I am supportive of the integration of complementary therapies into a cancer setting | 110 (94.8) | 20 (100) | 52 (98) | 15 (94) | 10 (91) |
| 2. I am confident discussing complementary therapies with patients | 61 (52.6) | 12 (60) | 31 (58) | 5 (31) | 5 (45) |
| 3. Many complementary therapies (for example, massage, yoga, acupuncture and mindfulness) have beneficial effects on psychological symptoms such as depression and anxiety and stress management | 113 (97.4) | 20 (100) | 52 (98) | 16 (100) | 11 (100) |
| 4. I feel I have sufficient knowledge about mind and body practices such as yoga, mindfulness, and therapies such as massage, reflexology and acupuncture to advise patients on benefits and contraindications | 42 (36.2) | 10 (50) | 17 (32) | 3 (19) | 3 (27) |
| 5. I feel I have sufficient knowledge about herbs and supplements to advise patients on benefits and contraindications | 24 (20.7) | 5 (25) | 7 (13) | 3 (19) | 6 (55) |
| 6. I believe complementary therapies can be beneficial to patients with cancer | 102 (88) | 20 (100) | 48 (91) | 14 (88) | 8 (73) |
| 7. I want to learn more about complementary therapies in cancer care | 104 (90) | 20 (100) | 51 (96) | 13 (81) | 10 (91) |
| 8. I have sufficient knowledge about medicinal use of cannabis to make recommendations to oncology patients | 12 (10.3) | 4 (24) | 2 (5) | 1 (8) | 4 (40) |
| 9. Health care professionals should receive continuing professional development about medicinal cannabis | 88 (93) | 17 (100) | 42 (98) | 9 (69) | 10 (100) |
| 10. There is sufficient scientific evidence supporting the efficacy of medicinal cannabis | 45 (47) | 7 (41) | 26 (60) | 8 (62) | 5(50) |
| 11. My attitude towards prescribing medical cannabis has changed | 31 (33) | 6 (35) | 16 (37) | 3 (23) | 3 (30) |
| 12. I am familiar with the endocannabinoid system | 17 (18) | 2 (12) | 3 (7) | 5 (38) | 3 (30) |
| **In your opinion or according to your experience, which of these cancer patient populations can benefit from medicinal cannabis:** | | | | | |
| 13. Patients with advanced disease receiving supportive care alone/end-of-life care | 55 (50.9) | 14 (70) | 24 (45) | 10 (62) | 3 (27) |
| 14. Patients receiving active disease-modifying treatment for advanced/metastatic cancer with refractory symptoms | 57 (49.1) | 12 (60) | 23 (43) | 11 (69) | 4 (36) |
| 15. Cancer survivors with persisting refractory (difficult to manage) symptoms | 51 (43.9) | 11 (55) | 21 (40) | 6 (38) | 6 (55) |
| 16.Early-stage patients with treatment-related refractory side effects or symptoms | 45 (38.7) | 9 (45) | 22 (42) | 7 (44) | 3 (27) |
| 17. Any patient with a cancer diagnosis (independent of symptom burden) | 26 (22.4) | 3 (15) | 17 (32) | 1 (6) | 3 (27) |
| 18. I don't know/cannot answer | 15 (12.9) | 3 (17) | 9 (17) | 1 (6) | 1 (9) |

^*Provides the numbers who ‘Agreed’ or ‘Strongly Agreed’ with the statements; total respondents n=116; occupation groups >10 respondents were included as distinct categories^

For the statements with substantial percentage differences among professions (2, 4 and 5) Fisher’s Exact test was carried out to test for differences by occupation, length of time at the hospital and age. For Statements 2 and 4 there was no significant difference among occupations. For Statement 5 (*I* *feel I have sufficient knowledge about herbs and supplements to advise patients on benefits and contraindications*) a larger percentage of pharmacists (p=0.008) compared to the other professions felt they had sufficient knowledge about herbs and supplements to advise patients on benefits and contraindications. Those working in the hospital for more than 12 months were more likely to agree with Statements 2 (p<0.001), 4 (p=0.038) and 5 (p=0.03). Older participants were also more likely to agree with Statements 2 (p=0.005), Statement 4 (p=0.006) and Statement 5 (p=0.024).

In the overall sample, the majority of participants wanted to learn more about each of the therapies included in the survey (Table 3). While knowledge on all types of CIM therapies desired, herbs (94/116) and dietary supplements (94/116) had slightly higher interest than other therapies. Few participants indicated already had enough knowledge or were (6/113) not being interested (3/116) in learning any further about CIM and lifestyle interventions.

Table 3 reports attitudes towards CIM and lifestyle medicine among HCPs. The majority (60.7%) of participants were undecided about whether they had seen patients improve faster when using CIM along with conventional health practices. Nearly all (89%) participants felt it was essential to network and build relationships with providers within the hospital. A third (33.6%) of participants agreed with feeling that their professional training had prepared them for integration of CIM and lifestyle medicine into their practice.

**Table 3.** Desire to learn more about CIM among participants

| I want to learn more about the benefits and contraindications for cancer patients of: | All respondents  n = 116 (%) | Allied Health  n=20 (%) | Nurse  n=53 (%) | Oncologist  n=16 (%) | Pharmacist  n=11 (%) |
| --- | --- | --- | --- | --- | --- |
| Dietary supplements | 94 (81) | 15 (75) | 46 (87) | 12 (75) | 10 (91) |
| Herbs | 94 (81) | 16 (80) | 47 (89) | 10 (62) | 9 (82) |
| Mind body therapies | 89 (77) | 16 (80) | 47 (89) | 10 (62) | 8 (73) |
| Nutrition | 87 (75) | 14 (70) | 44 (83) | 10 (62) | 8 (73) |
| Acupuncture | 86 (74) | 15 (75) | 47 (89) | 11 (69) | 6 (55) |
| Exercise | 80 (69) | 13 (65) | 43 (81) | 8 (50) | 7 (64) |
| Massage and reflexology | 80 (69) | 15 (75) | 45 (85) | 6 (38) | 6 (55) |
| Have enough knowledge about complementary therapies and lifestyle interventions | 6 (5) | 0 (0) | 1 (2) | 1 (6) | 0 (0) |
| Not interested in learning any further about complementary therapies and lifestyle interventions | 3 (3) | 1 (5%) | 1 (2%) | 2 (12%) | 0 (0) |
| I have seen patients improve faster when they used a complementary therapy along with conventional health practices | 39 (36) | 8 (42) | 22 (44) | 2 (13) | 3 (27) |
| I feel it is essential to network and build relationships with complementary therapies, exercise oncology and integrative oncology providers within the hospital | 95 (89) | 18 (95) | 47 (94) | 11 (73) | 10 (91) |
| I feel my professional training has prepared me for integration of complementary therapies and lifestyle medicine | 36 (34) | 10 (53) | 17 (34) | 1 (6.7 | 4 (36) |

We sought to determine whether any significant differences existed for each of these statements based on occupation, length of time working at the hospital and age. A minimal difference was found for occupation (p=0.045) while no effect was found for length of time at the hospital or age.

***Practice – CIM and MC recommendations, prescribing and referrals***

A total of 85 HCPs (75%) used CIM and lifestyle therapies to support their own health (Table 4), with 44% using massage/reflexology, nutrition (43%), exercise (64%) and dietary supplements (33%). CIM use was lowest among oncologists, although there was no statistical association between occupation and CIM use (Fisher’s exact test p=0.196). Few respondents reported using acupuncture (16%) or herbs (16%). Most HCPs (65%) would not advise against any of the individual CIM or against MC. Herbs (14%) and dietary supplements (7%) were the CIM therapies most likely to be advised against. Among the different occupations, more oncologists compared to the other occupations recommended against herbs (40%) and dietary supplements (27%), though numbers small overall. There was no association found between personal use of CIM and advice against CIM or MC use to cancer patients.

**Table 4.** Use of CIM, lifestyle and diet among HCPs, recommendations for and against

|  | All respondents  n=116 (%) | Nurses  n=53 (%) | Allied health  n=20 (%) | Oncologists  n=16 (%) | Pharmacists  n=11 (%) |
| --- | --- | --- | --- | --- | --- |
| Do you use complementary therapies, lifestyle and diet based therapies to support your own health? | 85 (75) | 40 (75) | 18 (90) | 9 (56) | 7 (64) |
| Acupuncture | 15 (13) | 4 (8) | 4 (20) | 1 (6) | 1 (9) |
| Dietary supplements | 38 (33) | 16 (30) | 8 (40) | 3 (19) | 5 (45%) |
| Exercise | 74 (64) | 16 (80) | 35 (66) | 8 (50) | 6 (55) |
| Herbs | 19 (16) | 8 (15) | 3 (15) | 1 (6) | 4 (36) |
| Massage and reflexology | 51 (44) | 24 (45) | 15 (75) | 1 (6) | 4 (36) |
| Mind body therapies | 43 (37) | 23 (43) | 9 (45) | 2 (12) | 5 (45) |
| Nutrition | 50 (43) | 24 (45) | 10 (50) | 5 (31) | 4 (36) |
| None of the above | 29 (25) | 13 (25) | 2 (10) | 7 (44) | 4 (36) |
| Which, if any, complementary therapies or lifestyle changes would you strongly advise against patient use? |  |  |  |  |  |
| Mind body therapies | 2 (2) | 0 (0) | (0) | 1 (6) | 0 (0) |
| Herbs | 16 (14) | 4 (8) | 2 (10) | 6 (38) | 2 (18) |
| Dietary supplements | 8 (7) | 2 (4) | 0 (0) | 4 (25) | 1 (9) |
| Massage & reflexology | 1 (1) | 0 (0) | 0 (0) | 1 (6) | 0 (0) |
| Acupuncture | 3 (3) | 2 (4) | 0 (0) | 0 (0) | 1 (9.1) |
| Exercise | 1 (1) | 0 (0) | 0 (0) | 1 (6) | 0 (0) |
| Nutrition | 2 (2) | 0 (0) | 0 (0) | 2 (2) | 0 (0) |
| Medicinal cannabis | 3 (3) | 0 (0) | 0 (0) | 2 (12) | 0 (0) |
| None of the above | 75 (65) | 40 (75) | 15 (75) | 7 (44) | 8 (73) |

Participants were asked who they recommended their patients seek advice about CIM from (Table 5). The highest rated was an integrative and supportive care medical specialist (31%), followed by an oncologist (18%). The majority of participants (63% yes definitely, 23% yes slightly) indicated that they would be more inclined to recommend or allow the use of some CIM if a pharmacy service existed which evaluated the potential interaction between herbs and supplements with cancer treatments.

**Table 5.** Seeking advice about CIM

| Who do you recommend your patients seek advice about CIM from? | Yes  n=294* (%) |
| --- | --- |
| Integrative and supportive care medical specialist | 82 (31) |
| Oncologist | 49 (18) |
| Dietitian | 40 (15) |
| Pharmacist | 29 (11) |
| GP | 28 (11) |
| Clinical nurse consultant | 27 (10) |
| No-one I discuss this with them | 11 (4.1) |
| **If there was a pharmacy service to evaluate the potential interaction between herbs and/or supplements with cancer treatments, would this make you more inclined to recommend or allow use of some of these therapies** | n = 116 |
| I don't know | 6 (5.6) |
| Will not affect my decision | 9 (8.4) |
| Yes definitely | 67 (63) |
| Yes slightly | 25 (23) |
| Unknown | 9 |

*More than one response permitted

A large percentage of HCPs (71%) indicated knowledge of their patients being prescribed medicinal cannabis (Online Resource 2). When asked about knowledge regarding self-prescribing of medicinal cannabis, slightly less than half (41%) were aware that their patients had been self-prescribing cannabis and a similar percentage did not know (46%). A small percentage (6.3%) were not aware whether their patients were self-prescribing cannabis. Patient experiences and medical literature were most influential in changing attitudes towards medicinal cannabis (Online Resource 2).

Participants were asked whether they agreed that they had sufficient knowledge to refer patients to the Integrative Oncology and Supportive Care Department at Chris O’Brien Lifehouse (Online Resource 2). Between approximately a third and two thirds of participants agreed that they had sufficient knowledge to refer patients to the service. Knowledge for referral was lowest for exercise physiology (34%) and survivorship program (38%). The level of agreement was similar between different occupations except for pharmacists, who tended to agree less than those in other occupations.

***Symptom management with medicinal cannabis***

Over half of the participants felt that medicinal cannabis may be helpful for all the cancer-related symptoms included in the survey (Online Resource 2).Cancer and treatment related nausea and vomiting were the most common symptom HCPS agreed MC may be beneficial, 84.2% selecting cancer related nausea and 83.2% selecting chemo-related nausea/vomiting.

The majority of participants identified driving impairment (63.2%) and drowsiness (66.3%) as a major side effect of MC. For most side effects, health care professionals neither agreed nor disagreed (Figure 1).

**Figure 1.** Agreement of HCP’s with the major side effects of medicinal cannabis
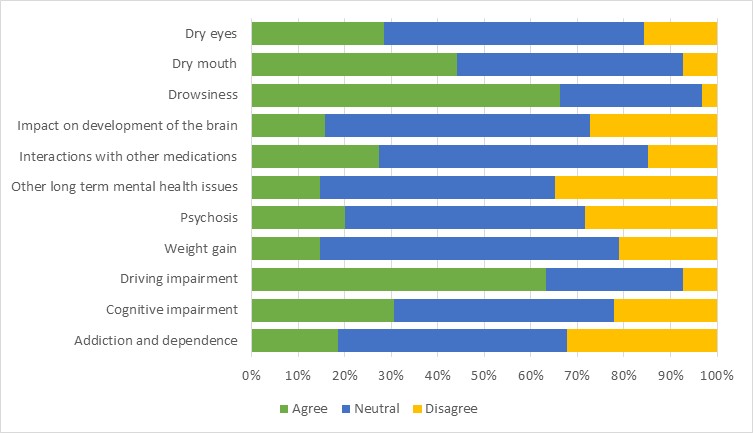


**Discussion**

Our study found that nearly all 116 respondents supported the integration of complementary and integrative medicine (CIM) into cancer care, and believed these therapies can be beneficial to patients with cancer but the majority of respondents did not feel they had adequate knowledge to advise patients on CIM. For medicinal cannabis (MC), attitudes were more ambiguous, with only half of respondents agreeing that there was adequate evidence for the efficacy of MC, or that MC was beneficial to people with cancer. Nearly all respondents wanted to learn more about complementary therapies, this is consistent with other studies [23]. Only 33% of health care professionals’ felt prepared to integrate CIM into their work. As hypothesised, HCPs knowledge of individual CIM therapies varied between mind body, and herbs and supplements.

The majority of respondents agreed CIM beneficial effects for people with cancer. Nearly all respondents believed CIM was beneficial to people with cancer, and mind-body therapies had beneficial effects on psychological symptoms such as depression and anxiety and stress management. For MC may benefit cancer related symptoms, and this was higher for symptoms such as managing cancer and treatment related nausea, and appetite. Side effects of MC were thought to be primarily driving impairment and drowsiness but there was uncertainty around other impacts.

With only a few exceptions, knowledge and attitudes to CIM and MC in our study were not influenced by age, gender, occupation or length of time at the hospital. Those respondents who had worked at the hospital longer and were older, were more likely to report confidence in discussing CIM with patients, and reported having sufficient knowledge about mind body practices and herbs and supplements.

Knowledge gaps were highest for MC, with only 10% reporting they had adequate knowledge to recommend or 17% were familiar with the endocannabinoid system. Only 20% of HCPs were confident advising on the benefits and contraindications of herbs and supplements, confidence was slightly higher for mind-body practices (36%). All HCPs wanted to learn more about the benefits of CIM and MC. Interest was highest for dietary supplements (81%) and herbs (81%), with the majority of oncologists wanting to learn about the benefits and contraindications of dietary supplements, and herbs.

Despite limited confidence in benefits and contraindications of herbs and supplements, only 7-14% of the HCPs would advise against the use of herbs and dietary supplements. In a pooled prevalence of studies of people with cancer, 22% used herbal medicine and this is higher in certain population groups such as women with breast cancer where 41% reported use of herbal medicine [4, 33]. Dietary supplement use is higher, with studies indicating use by almost one in two people with cancer (including those receiving cancer treatment), with a slightly lower but significant (36%) level of consumption in men [19, 25, 39]. Despite this prevalence of use in Australian cancer patients, respondents in our study did not feel they were equipped to advise on herbs and supplements, and this area received the highest interest by HCPs in learning more, compared to mind-body therapies.

Advice regarding the safe use of herbal medicine or dietary supplement use during cancer treatment is an important part of comprehensive cancer care. Yet only 16 cancer services in Australia have dedicated healthcare practitioners providing advice on the use of any CIM [36]. In our study, there was strong support for a pharmacy service to advise on the potential interaction and safety of herbs and/or supplements with cancer treatments. The majority of respondents stated this would make them more likely to recommend or allow use. More than half of the pharmacists in our survey reported that they were confident in advising on herbs and supplements. For CIM in general, respondents in our study were most likely to refer patients to the integrative and supportive care specialist within the hospital. Taken together, these findings indicate that the safety and choices of people with cancer and their supportive care can be greatly enhanced by providing a level of CIM and MC specialist knowledge within a comprehensive cancer setting.

Meeting cancer patient expectations, cultural preferences, beliefs and information needs improves patient outcomes [37]. One in two Australians with cancer use CIM, it aligns with their personal values, beliefs and cultural identity [18]. People use CIM to help cope with the side effects of conventional cancer treatments, improve survival and long-term outcomes, and to support their mental health, wellbeing, weight management, self-efficacy, and quality of life throughout the cancer continuum [1, 11, 13, 34]. Additionally, patients receiving treatment at an institution that supports an IO program may have improved survival [10, 18, 34, 38]. Our survey, in line with other studies, shows that the education, integration, pathways and translation of evidence into practice is a major barrier to preferences being expressed by HCPs and people with cancer[24].

The establishment of designated cancer treatment centres in Australia with links to rural and remote centres may provide the basis for speciality advice on integrative oncology to patients and practitioners. Similar to the program of National Cancer Institute designated cancer centres in the US that have developed or are developing integrative oncology programs to assist, along with guidelines to support providers who participate in these programs [27, 45]. This would require the training of health care professionals in integrative oncology, competencies and training options are emerging [41, 44].

Whilst knowledge and attitudes of general practitioners to MC have been assessed [20], no studies conducted in Australia have explored attitudes of oncology HCPs. The findings in our study were similar to other studies conducted in Europe, where oncology healthcare professionals increasingly agree that MC reduces patient suffering, and has benefits, particularly in people with advanced cancer [12].

The study was conducted at a hospital that has provided a range of CIM alongside conventional cancer care since it opened in 2013, and more recently prescription, of MC. Through this exposure, we anticipated that the HCPs surveyed may have different knowledge and attitudes toward CIM compared to participants in other surveys. In other surveys 58%-90% of HCPs reported having inadequate knowledge to answer questions about CIM, compared to 51% of HCPs in our study reporting that they felt confident in discussing CIM with patients[24]. However, this knowledge did not extend to side effects of MC, or the endocannabinoid system and most respondents did not have sufficient knowledge to make recommendations about MC use to people with cancer.

**Limitations:**

Our study had several limitations. We used convenience sampling from a single institution, and this may impact external validity. Participation in the survey was voluntary. However, the sample may not have been representative of the hospital population resulting in selection bias. The survey was administered in an anonymous and confidential manner which may mitigate some bias. There are no validated tools for measuring attitudes and beliefs to the use of cannabis in cancer care which may have resulted in information bias.

**Conclusion**

The uptake and integration of evidence-based and informed CIM and MC by oncologists and other HCPs in cancer care is hampered by a lack of knowledge of benefits and contraindications, gaps in education and training and the lack of adequate referral pathways [28]. The results of this survey will inform the development of ongoing education activities, knowledge sharing and research activities. Effective and safe integration of CIM and MC may require the targeted development of services such as pharmacy to evaluate the safety of herbs and supplements with a focus on drug-herb interactions, and inclusion of cancer specialists who have received specific training in CIM and MC [21]. The targeted development of pharmacy and training of dedicated HCPs to provide advice on CIM and MC would support informing the choice of 1 in 2 Australians with cancer who use CIM.

**References**

1. Amichai T, Grossman M, Richard M (2012) Lung cancer patients’ beliefs about complementary and alternative medicine in the promotion of their wellness European Journal of Oncology Nursing 16: 520-527

2. Arnfinsen JL, Kisa A (2021) Assessment of Norwegian physicians’ knowledge, experience and attitudes towards medical cannabis Drugs: Education, Prevention and Policy 28: 165-171 doi:10.1080/09687637.2020.1806208

3. Arthur K, Belliard JC, Hardin SB, Knecht K, Chen C-S, Montgomery SJC, oncology c (2013) Reasons to use and disclose use of complementary medicine use–an insight from cancer patients 2: 81

4. Asiimwe JB, Nagendrappa PB, Atukunda EC, Kamatenesi MM, Nambozi G, Tolo CU, Ogwang PE, Sarki AM (2021) Prevalence of the Use of Herbal Medicines among Patients with Cancer: A Systematic Review and Meta-Analysis Evidence-Based Complementary and Alternative Medicine 2021: 9963038 doi:10.1155/2021/9963038

5. Bar-Lev Schleider L, Mechoulam R, Lederman V, Hilou M, Lencovsky O, Betzalel O, Shbiro L, Novack V (2018) Prospective analysis of safety and efficacy of medical cannabis in large unselected population of patients with cancer European Journal of Internal Medicine 49: 37-43 doi:10.1016/j.ejim.2018.01.023

6. Berger CC, Johnson KF (2017) Complementary and Integrative Health Assessment for Practitioners Scale: Initial Development and Validation Journal of Mental Health Counseling 39: 305-319 doi:10.17744/mehc.39.4.03

7. Bocock C, Reeder AI, Perez D, Trevena J (2011) Beliefs of New Zealand doctors about integrative medicine for cancer treatment Integrative cancer therapies 10: 280-288

8. Braun IM, Wright A, Peteet J, Meyer FL, Yuppa DP, Bolcic-Jankovic D, LeBlanc J, Chang Y, Yu L, Nayak MM, Tulsky JA, Suzuki J, Nabati L, Campbell EG (2018) Medical Oncologists' Beliefs, Practices, and Knowledge Regarding Marijuana Used Therapeutically: A Nationally Representative Survey Study Journal of clinical oncology : official journal of the American Society of Clinical Oncology 36: 1957-1962 doi:10.1200/JCO.2017.76.1221

9. Christina J, Abigail W, Cuthbertson LA (2016) Nurses’ Knowledge and Attitudes toward Complementary Therapies for Cancer: A Review of the Literature Asia-Pacific Journal of Oncology Nursing 3: 241-251 doi:10.4103/2347-5625.189816

10. Crudup T, Li L, Dorr JW, Lawson E, Stout R, Niknam PV, Jones J, Steen RG, Casner S, Lu LL, Wang Y, Scott J, Zanine S, Robertshaw S, Broderick G, Singh S, Lu J, Zhou L, Palommella V, Harris T, Hanamirian M, Reddy MS, Cowgill B, Rice J, Nagaraja A, Jonas W (2021) Breast Cancer Survivorship and Level of Institutional Involvement Utilizing Integrative Oncology Journal of Oncology 2021: 4746712 doi:10.1155/2021/4746712

11. Ee C, Cave AE, Naidoo D, Boyages J (2019) Prevalence of and attitudes towards complementary therapy use for weight after breast cancer in Australia: a national survey BMC complementary and alternative medicine 19: 332 doi:10.1186/s12906-019-2747-6

12. Filetti M, Trapani D, Cortellini A, Cofini V, Necozione S, Pinato DJ, Porzio G, Marchetti P, Giusti R (2021) Knowledge and attitudes of Italian medical oncologists and palliative care physicians toward medical use of cannabis in cancer care: a national survey Support Care Cancer 29: 7845-7854 doi:10.1007/s00520-021-06383-7

13. Gall A, Anderson K, Diaz A, Matthews V, Adams J, Taylor T, Garvey G (2019) Exploring traditional and complementary medicine use by Indigenous Australian women undergoing gynaecological cancer investigations Complementary Therapies in Clinical Practice 36: 88-93 doi:<https://doi.org/10.1016/j.ctcp.2019.06.005>

14. Ghaferi AA, Schwartz TA, Pawlik TM (2021) STROBE Reporting Guidelines for Observational Studies JAMA Surg 156: 577-578 doi:10.1001/jamasurg.2021.0528

15. Grant SJ, Marthick M, Lacey J (2018) Establishing an integrative oncology service in the Australian healthcare setting—the Chris O’Brien Lifehouse Hospital experience Support Care Cancer: 1-8

16. Harnett J, Le TQ, Smith L, Krass I (2018) Perceptions, opinions and knowledge of pharmacists towards the use of complementary medicines by people living with cancer Int J Clin Pharm 40: 1272-1280 doi:10.1007/s11096-018-0645-5

17. Hewa-Gamage D, Blaschke S, Drosdowsky A, Koproski T, Braun A, Ellen S (2019) A Cross-sectional Survey of Health Professionals' Attitudes toward Medicinal Cannabis Use as Part of Cancer Management J Law Med 26: 815-824

18. Hunter J, Ussher J, Parton C, Kellett A, Smith C, Delaney G, Oyston E (2018) Australian integrative oncology services: a mixed-method study exploring the views of cancer survivors BMC complementary and alternative medicine 18: 153 doi:10.1186/s12906-018-2209-6

19. Jung AY, Cai X, Thoene K, Obi N, Jaskulski S, Behrens S, Flesch-Janys D, Chang-Claude J (2019) Antioxidant supplementation and breast cancer prognosis in postmenopausal women undergoing chemotherapy and radiation therapy The American journal of clinical nutrition 109: 69-78 doi:10.1093/ajcn/nqy223

20. Karanges EA, Suraev A, Elias N, Manocha R, McGregor IS (2018) Knowledge and attitudes of Australian general practitioners towards medicinal cannabis: a cross-sectional survey BMJ Open 8: e022101 doi:10.1136/bmjopen-2018-022101

21. Karim S, Benn R, Carlson LE, Fouladbakhsh J, Greenlee H, Harris R, Henry NL, Jolly S, Mayhew S, Spratke L, Walker EM, Zebrack B, Zick SM (2021) Integrative Oncology Education: An Emerging Competency for Oncology Providers Current oncology (Toronto, Ont) 28: 853-862 doi:10.3390/curroncol28010084

22. Keene MR, Heslop IM, Sabesan SS, Glass BD (2019) Complementary and alternative medicine use in cancer: A systematic review Complementary Therapies in Clinical Practice 35: 33-47 doi:<https://doi.org/10.1016/j.ctcp.2019.01.004>

23. Keene MR, Heslop IM, Sabesan SS, Glass BD (2020) Knowledge, attitudes and practices of health professionals toward complementary and alternative medicine in cancer care – a systematic review Journal of Communication in Healthcare 13: 205-218 doi:10.1080/17538068.2020.1755202

24. Keene MR, Heslop IM, Sabesan SS, Glass BD (2022) Knowledge, attitudes, and practices of Australian oncology health professionals on complementary medicines Journal of Pharmacy Practice and Research n/a doi:<https://doi.org/10.1002/jppr.1838>

25. Klafke N, Eliott JA, Wittert GA, Olver IN (2012) Prevalence and predictors of complementary and alternative medicine (CAM) use by men in Australian cancer outpatient services Annals of Oncology 23: 1571-1578 doi:10.1093/annonc/mdr521

26. Lintzeris N, Mills L, Suraev A, Bravo M, Arkell T, Arnold JC, Benson MJ, McGregor IS (2020) Medical cannabis use in the Australian community following introduction of legal access: the 2018–2019 Online Cross-Sectional Cannabis as Medicine Survey (CAMS-18) Harm Reduction Journal 17: 37 doi:10.1186/s12954-020-00377-0

27. Mao JJ, Ismaila N, Bao T, Barton D, Ben-Arye E, Garland EL, Greenlee H, Leblanc T, Lee RT, Lopez AM, Loprinzi C, Lyman GH, MacLeod J, Master VA, Ramchandran K, Wagner LI, Walker EM, Bruner DW, Witt CM, Bruera E (2022) Integrative Medicine for Pain Management in Oncology: Society for Integrative Oncology–ASCO Guideline Journal of Clinical Oncology: JCO.22.01357 doi:10.1200/JCO.22.01357

28. Newell S, Sanson-Fisher RW (2000) Australian oncologists' self-reported knowledge and attitudes about non-traditional therapies used by cancer patients The Medical journal of Australia 172: 110-113

29. Oliver SJ (2013) The role of traditional medicine practice in primary health care within Aboriginal Australia: a review of the literature Journal of ethnobiology and ethnomedicine 9: 46 doi:10.1186/1746-4269-9-46

30. Oskay-Özcelik G, Lehmacher W, Könsgen D, Christ H, Kaufmann M, Lichtenegger W, Bamberg M, Wallwiener D, Overkamp F, Diedrich KJAoo (2007) Breast cancer patients' expectations in respect of the physician–patient relationship and treatment management results of a survey of 617 patients 18: 479-484

31. Roberts CS, Baker F, Hann D, Runfola J, Witt C, McDonald J, Livingston ML, Ruiterman J, Ampela R, Kaw OC (2006) Patient-physician communication regarding use of complementary therapies during cancer treatment Journal of Psychosocial Oncology 23: 35-60

32. Roter DL, Yost KJ, O’Byrne T, Branda M, Leppin A, Kimball B, Fernandez C, Jatoi A, Kumbamu A, Montori V (2016) Communication predictors and consequences of complementary and alternative medicine (CAM) discussions in oncology visits Patient Educ Couns 99: 1519-1525

33. Samuels N, Ben-Arye E, Maimon Y, Berger R (2017) Unmonitored use of herbal medicine by patients with breast cancer: reframing expectations Journal of cancer research and clinical oncology 143: 2267-2273 doi:10.1007/s00432-017-2471-x

34. Segev Y, Lavie O, Stein N, Saliba W, Samuels N, Shalabna E, Raz OG, Schiff E, Ben-Arye E (2021) Correlation between an integrative oncology treatment program and survival in patients with advanced gynecological cancer Support Care Cancer 29: 4055-4064 doi:10.1007/s00520-020-05961-5

35. Shalom-Sharabi I, Lavie O, Samuels N, Keinan-Boker L, Lev E, Ben-Arye EJJoCR, Oncology C (2017) Can complementary medicine increase adherence to chemotherapy dosing protocol? A controlled study in an integrative oncology setting 143: 2535-2543

36. Smith CA, Hunter J, Delaney GP, Ussher JM, Templeman K, Grant S, Oyston E (2018) Integrative oncology and complementary medicine cancer services in Australia: findings from a national cross-sectional survey BMC Complementary & Alternative Medicine 18: 289

37. Stie M, Jensen LH, Delmar C, Norgaard B (2020) Open dialogue about complementary and alternative medicine (CAM) integrated in conventional oncology care, characteristics and impact. A systematic review Patient Educ Couns 103: 2224-2234 doi:10.1016/j.pec.2020.06.003

38. Stomski NJ, Petterson A, Kristjanson L, Lobb EA, Phillips M, Williams A, Morrison P, Joske D (2018) The effect of self-selected complementary therapies on cancer patients’ quality of life and symptom distress: A prospective cohort study in an integrative oncology setting Complementary Therapies in Medicine 37: 1-5 doi:<https://doi.org/10.1016/j.ctim.2018.01.006>

39. Tank M, Franz K, Cereda E, Norman K (2021) Dietary supplement use in ambulatory cancer patients: a survey on prevalence, motivation and attitudes Journal of cancer research and clinical oncology 147: 1917-1925 doi:10.1007/s00432-021-03594-7

40. Tasaki K, Maskarinec G, Shumay DM, Tatsumura Y, Kakai H (2002) Communication between physicians and cancer patients about complementary and alternative medicine: exploring patients' perspectives Psycho‐Oncology 11: 212-220

41. Thomae AV, Rogge AA, Helmer SM, Icke K, Witt CM (2022) Development, Implementation, and Evaluation of an e-Learning in Integrative Oncology for Physicians and Students Involving Experts and Learners: Experiences and Recommendations Journal of cancer education : the official journal of the American Association for Cancer Education: 1-8 doi:10.1007/s13187-022-02189-1

42. Wilson A, Davis C (2022) Attitudes of Cancer Patients to Medicinal Cannabis Use: A Qualitative Study Australian Social Work 75: 192-204 doi:10.1080/0312407X.2021.1904264

43. Witt CM, Balneaves LG, Cardoso MJ, Cohen L, Greenlee H, Johnstone P, Kucuk O, Mailman J, Mao JJ (2017) A Comprehensive Definition for Integrative Oncology J Natl Cancer Inst Monogr 2017 doi:10.1093/jncimonographs/lgx012

44. Witt CM, Balneaves LG, Carlson LE, Cohen M, Deng G, Fouladbakhsh JM, Kinney AY, Mehta A, Mailman J, Pole L (2022) Education competencies for integrative oncology—results of a systematic review and an international and interprofessional consensus procedure Journal of Cancer Education 37: 499-507

45. Yun H, Sun L, Mao JJ (2017) Growth of Integrative Medicine at Leading Cancer Centers Between 2009 and 2016: A Systematic Analysis of NCI-Designated Comprehensive Cancer Center Websites J Natl Cancer Inst Monogr 2017 doi:10.1093/jncimonographs/lgx004

**Statements and Declarations**

**Funding:** This research and the patient treatments were supported by a donation from the McNiven family and Mostyn family. The funders had no role in study design, data collection and analysis, decision to publish, or preparation of the manuscript.

**Competing interests**

The authors declare no competing interests.

**Author Contributions:** All authors contributed to the study conception and design. Material preparation, data collection and analysis were performed by Suzanne Grant, and Maria Gonzalez. The first draft of the manuscript was written by Suzanne Grant and all authors commented on previous versions of the manuscript. All authors read and approved the final manuscript.

**Ethics approval**

The study received ethics approval from the Sydney Local Area Health District Ethics Committee in May 2019 (HREC/18/RPAH/519). All procedures performed in studies involving human participants were in accordance with the ethical standards of the institutional and/or national research committee and with the 1964 Helsinki Declaration and its later amendments or comparable ethical standards.

**Consent to participate**

Informed consent was obtained from all individual participants included in the study.

**Consent for publication**

All authors confirm that human research participants provided informed consent for publications.
