# Supplementary material for: Knowledge and attitudes towards medicinal cannabis and complementary and integrative medicine (CIM): a survey of healthcare professionals working in a cancer hospital in Australia
Source: Support Care Cancer. 2023 Oct 11;31(11):623. doi: 10.1007/s00520-023-08080-z (PMC10567955; doi:10.1007/s00520-023-08080-z)
Supplement: Supplementary file 2 — ESM 2 [file 520_2023_8080_MOESM2_ESM.docx]

Introduction
Dear Healthcare Professional,
 
We are interested in your attitudes and beliefs around **complementary therapies**, **lifestyle medicine** and the use of **medicinal cannabis** in cancer care. There are no right or wrong answers to any time, please response to each according to how you feel about complementary therapies, lifestyle medicine and medicinal cannabis.
 
***Complementary therapies*** are defined as a group of diverse medical and health care interventions, practices, products or disciplines that are not generally part of conventional medicine. This includes natural products (such as herbs, vitamins and minerals) or mind and body practices (yoga, mindfulness, massage, qi gong, tai chi and so on).
 
***Integrative oncology*** is a patient-centered, evidence-informed field of comprehensive cancer care that uses mind-body practices, natural products, and lifestyle modifications from different traditions alongside conventional cancer treatments.
 
All your answers are **not identifiable**, will be **kept confidential** and will help us to design to future education sessions. Participation is voluntary.
 
The survey will take approximately 8 minutes to complete.

 Click to read the [Participant Information Sheet](https://lifehouse.qualtrics.com/CP/File.php?F=F_bk1m5yCbH8w59oa).

Consent
Please click the button below to consent to continue. Completion of the survey will be taken as your consent to participate in this study.

- I consent

Q1. Where do you work?

- Inpatient wards
- Radiation oncology
- Day therapy
- Surgical theaters
- Outpatient clinics
- Pharmacy
- Prefer not to say
- Other (please specify) ____________________________________________

Q2. Are you a:

- Surgeon
- Medical oncologist
- Radiation oncologist
- Nurse
- Allied health professional
- Pharmacist
- Medical trainee or fellow
- Prefer not to say
- Other (please specify) __________________________________________________

Q3. Please rate your level of agreement with the following statements:

|  | Strongly agree | Agree | Undecided | Disagree | Strongly disagree |
| --- | --- | --- | --- | --- | --- |
| I am supportive of the integration of complementary therapies into a cancer setting |  |  |  |  |  |
| I am confident discussing complementary therapies with patients |  |  |  |  |  |
| Many complementary therapies (for example massage, yoga, acupuncture and mindfulness) have beneficial effects on psychological symptoms such as depression and anxiety and stress management |  |  |  |  |  |
| I feel I have sufficient knowledge about **mind and body** practices such as yoga, mindfulness, and therapies such as massage, reflexology and acupuncture to advise patients on benefits and contraindications |  |  |  |  |  |
| I feel I have sufficient knowledge about **herbs and supplements** to advise patients on benefits and contraindications |  |  |  |  |  |
| I believe complementary therapies can be beneficial to patients with cancer |  |  |  |  |  |
| I want to learn more about complementary therapies in cancer care |  |  |  |  |  |

Q4. I want to learn more about the benefits and contraindications for cancer patients of:
*Please check more than one box if applicable*

- Mind body therapies (e.g. mindfulness, yoga, qi gong, tai chi)
- Herbs
- Dietary supplements
- Massage and reflexology
- Acupuncture
- Exercise
- Nutrition
- All of the above
- I feel I have enough knowledge about complementary therapies and lifestyle interventions
- I am not interested in learning any further about complementary therapies and lifestyle interventions
- Other (please specify) ______________________________________________

Q5. I use complementary therapies and lifestyle and diet based therapies to support my own health

- Yes
- No

Q5.1. Which ones
 *Please check more than one box if applicable*

- Mind body therapies (e.g. mindfulness, yoga, qi gong, tai chi)
- Herbs
- Dietary supplements
- Massage and reflexology
- Acupuncture
- Exercise
- Nutrition
- Other (please specify) ______________________________________________

Q6. I have recommended complementary therapies and lifestyle, exercise and/or nutrition advice to my patients
 *These therapies combined with conventional cancer care is known as Integrative Oncology*

- Yes
- No

Q6.1. Which ones
 *Please check more than one box if applicable*

- Mind body therapies (e.g. mindfulness, yoga, qi gong, tai chi)
- Herbs
- Dietary supplements
- Massage and reflexology
- Acupuncture
- Exercise
- Nutrition
- Medicinal cannabis
- Other (please specify) _____________________________________________

Q7. Which, if any, complementary therapies or lifestyle changes would you strongly advise against patient use?
*Please check more than one box if applicable*

- Mind body therapies (e.g. mindfulness, yoga, qi gong, tai chi)
- Herbs
- Dietary supplements
- Massage and reflexology
- Acupuncture
- Exercise
- Nutrition
- Medicinal cannabis
- None of the above
- Other (please specify) ______________________________________________

Q8. Please rate your level of agreement with the following statements:

|  | Strongly agree | Agree | Undecided | Disagree | Strongly disagree |
| --- | --- | --- | --- | --- | --- |
| I have seen patients **improve faster** when they used a complementary therapy along with conventional health practices |  |  |  |  |  |
| I feel it is essential to **network and build relationships** with complementary therapies, exercise oncology and integrative oncology providers within the hospital |  |  |  |  |  |
| I feel my **professional training has prepared me** for integration of complementary therapies and lifestyle medicine |  |  |  |  |  |

Q9. Who do you recommend your patients seek advice about complementary therapies from?
*Please check more than one box if applicable*

- No-one I discuss this with them
- Pharmacist
- Clinical nurse consultant
- GP
- Dietitian
- Oncologist
- Integrative and supportive care medical specialist
- Other (please specify) _____________________________________________

Q10. If there was a pharmacy service to evaluate the potential interaction between herbs and/or supplements with cancer treatments, would this make you more inclined to recommend or allow use of some of these therapies?

- Yes definitely
- Yes slightly
- Will not affect my decision
- I don't know

**Medicinal cannabis**
This section explores current attitudes of healthcare professionals to the prescribing and use of **medicinal cannabis** in cancer care.
 
The use of medicinal cannabis to treat those suffering with cancer has become more widespread among the medical community and is moving to becoming a mainstream pharmacological treatment. However, healthcare professionals often hold different attitudes, experiences, and knowledge towards the topic.

Q11. In your opinion or according to your experience, which of these cancer patient populations can **benefit** from medicinal cannabis?
*Please check more than one box if applicable*

- Early-stage patients with treatment-related refractory side effects or symptoms (e.g. nausea, pain)
- Patients receiving active disease-modifying treatment for advanced/metastatic cancer with refractory symptoms
- Patients with advanced disease receiving supportive care alone/end-of-life care
- Cancer survivors with persisting refractory (difficult to manage) symptoms
- Any patient with a cancer diagnosis (independent of symptom burden)
- None of the above
- I don't know/cannot answer

Q12. Do you think medicinal cannabis may be **helpful** in managing the following cancer-related symptoms?

|  | Helpful | Somewhat helpful | Not helpful | I don't know |
| --- | --- | --- | --- | --- |
| Chemo-related nausea/vomiting |  |  |  |  |
| Cancer related nausea |  |  |  |  |
| Poor appetite |  |  |  |  |
| Depression |  |  |  |  |
| Anxiety |  |  |  |  |
| Sleep disturbances |  |  |  |  |
| General coping |  |  |  |  |
| Pain |  |  |  |  |
| Other (please specify) |  |  |  |  |

Q13. The major **side effects** of medicinal cannabis include:

|  | Strongly agree | Agree | Neutral | Disagree | Strongly disagree |
| --- | --- | --- | --- | --- | --- |
| Addiction and dependence |  |  |  |  |  |
| Cognitive impairment |  |  |  |  |  |
| Driving impairment |  |  |  |  |  |
| Weight gain |  |  |  |  |  |
| Psychosis |  |  |  |  |  |
| Other long-term mental health issues |  |  |  |  |  |
| Interactions with other medications |  |  |  |  |  |
| Impact on development of the brain |  |  |  |  |  |
| Drowsiness |  |  |  |  |  |
| Dry mouth |  |  |  |  |  |
| Dry eyes |  |  |  |  |  |
| Other (please specify) |  |  |  |  |  |

Q14. Please rate your level of agreement with the following statements:

|  | Strongly agree | Agree | Neutral | Disagree | Strongly disagree |
| --- | --- | --- | --- | --- | --- |
| I have sufficient knowledge about medicinal use of cannabis to make recommendations to oncology patients |  |  |  |  |  |
| Health care professionals should receive continuing professional development about medicinal cannabis |  |  |  |  |  |
| There is sufficient scientific evidence supporting the efficacy of medicinal cannabis |  |  |  |  |  |
| My attitude towards prescribing medical cannabis has changed |  |  |  |  |  |
| I am familiar with the endocannabinoid system |  |  |  |  |  |

Q15. In your opinion what has influenced your attitudes towards medicinal cannabis?
*Please check more than one box if applicable*

- Medical literature
- Experiences with patients
- News/Media
- Other physicians
- Lectures/Seminars
- Friends/Family
- Other (please specify) _____________________________________________

Q16. To your knowledge have your patients been prescribed medicinal cannabis?

- Yes
- No
- I don't know
- Not applicable

Q17. To your knowledge have your patients been self-prescribing medicinal cannabis?

- Yes
- No
- I don't know
- Not applicable

Q18. I have **sufficient knowledge** to refer patients to living room services (integrative oncology and supportive care) at Chris O’Brien Lifehouse. Please rate your level of agreement:

|  | Strongly agree | Agree | Neutral | Disagree | Strongly disagree |
| --- | --- | --- | --- | --- | --- |
| Acupuncture |  |  |  |  |  |
| Exercise physiology |  |  |  |  |  |
| Integrative oncology medical consultations |  |  |  |  |  |
| Oncology massage |  |  |  |  |  |
| Lymphoedema treatment |  |  |  |  |  |
| Mindfulness |  |  |  |  |  |
| Nurse consultant |  |  |  |  |  |
| Pelvic floor physiotherapy |  |  |  |  |  |
| Reflexology |  |  |  |  |  |
| Survivorship program |  |  |  |  |  |
| Yoga |  |  |  |  |  |
| Physiotherapy |  |  |  |  |  |

Q19. Did you know that acupuncture and reflexology is available for free in day therapy?

- Yes
- No

Q20. Did you know that massage and reflexology is available free for ward patients?

- Yes
- No

Q21. Did you know that inpatients can receive massage, mindfulness sessions, chair yoga, acupuncture and reflexology in their rooms on request?

- Yes
- No

Q22. Do you refer patients to the Living Room services?

- Yes
- No

Q23. Do you have any further comments you would like to add about the Living Room or complementary therapies?

________________________________________________________________

________________________________________________________________

________________________________________________________________

________________________________________________________________

________________________________________________________________

Q24. What is your gender?

- Male
- Female
- Prefer not to say

Q25. What is your age?

- <31
- 31-50
- >51
- Prefer not to say

Q26. Length of time at the hospital

- Less than 12 months
- More than 12 months
