# Supplementary material for: Knowledge and attitudes towards medicinal cannabis and complementary and integrative medicine (CIM): a survey of healthcare professionals working in a cancer hospital in Australia
Source: Support Care Cancer. 2023 Oct 11;31(11):623. doi: 10.1007/s00520-023-08080-z (PMC10567955; doi:10.1007/s00520-023-08080-z)
Supplement: Supplementary file 3 — ESM 3 [file 520_2023_8080_MOESM3_ESM.docx]

**Online Resource 2**

**Table 1.** Association between personal use and recommendations.

| I have recommended complementary therapies and lifestyle, exercise and/or nutrition advice to my patients | | | | |
| --- | --- | --- | --- | --- |
|  | No | Yes | Total | p-value^1^ |
| Use complementary therapies and lifestyle and diet based therapies to support my own health |  |  |  | >0.9 |
| No | 10 (36%) | 18 (64%) | 28 (100%) |  |
| Yes | 30 (37%) | 52 (63%) | 82 (100%) |  |
| **Total** | 40 (36%) | 70 (64%) | 110 (100%) |  |
| ^1^Pearson's Chi-squared test | | | | |

**Table 2.** HCP’s knowledge of their patients’ medicinal cannabis use

|  | n = 116^1^ |
| --- | --- |
| **To your knowledge, have your patients been prescribed medicinal cannabis?** | |
| I don't know | 20 (21%) |
| No | 2 (2.1%) |
| Not applicable | 6 (6.3%) |
| Yes | 67 (71%) |
| Unknown | 21 |
| **To your knowledge, have your patients been self-prescribing medicinal cannabis?** | |
| I don't know | 44 (46%) |
| No | 6 (6.3%) |
| Not applicable | 6 (6.3%) |
| Yes | 39 (41%) |
| Unknown | 21 |

**Table 3.** Influences on attitudes to medicinal cannabis

| **Source** | N = 253^1^ |
| --- | --- |
|  |  |
| Experiences with patients | 71 (31%) |
| Friends/Family | 16 (7.0%) |
| Lectures/Seminars | 12 (5.2%) |
| Medical literature | 56 (24%) |
| News/Media | 35 (15%) |
| Other physicians | 40 (17%) |

**Table 4.** Knowledge of Integrative Oncology for internal referral

| I have sufficient knowledge to refer patients to integrative oncology and supportive care* at Chris O’Brien Lifehouse. | Total agree  n (%) | Nurses  n (%) | Allied health  n (%) | Oncologists  n (%) | Pharmacists  n (%) |
| --- | --- | --- | --- | --- | --- |
| Acupuncture | 43 (45.7) | 17 (40) | 11 (65) | 5 (42) | 1 (10) |
| Exercise physiology | 32 (34) | 21 (49) | 12 (76) | 10 (83) | 1 (10) |
| Integrative oncology medical consultation | 51 (54.3) | 18 (42) | 8 (47) | 10 (83) | 2 (20) |
| Oncology massage | 56 (59.6) | 27 (63) | 14 (82) | 7 (58) | 1 (10) |
| Lymphoedema treatment | 47 (50) | 23 (53) | 13 (76) | 9 (75) | 1 (10) |
| Mindfulness | 57 (60.6) | 28 (65) | 13 (76) | 7 (58) | 4 (40) |
| Nurse consultant | 56 (59.6) | 23 (53) | 9 (53) | 8 (67) | 4 (40) |
| Pelvic floor physiotherapy | 60 (63.8) | 10 (23) | 12 (71) | 5 (42) | 2 (20) |
| Reflexology | 52 (55.3) | 24 (56) | 11 (65) | 3 (25) | 1 (10) |
| Survivorship program | 36 (38.3) | 8 (19) | 7 (41) | 6 (50) | 3 (75) |
| Yoga | 48 (51.1) | 21 (49) | 13 (76) | 6 (50) | 3 (30) |

*known as the Living Room

**Table 5.** Symptom Management and Medicinal cannabis

| Do you think medicinal cannabis may be helpful for managing the following cancer-related symptoms? |  |  |  |  |  |
| --- | --- | --- | --- | --- | --- |
| Cancer related nausea | 80 (84) |  |  |  |  |
| Chemo-related nausea/vomiting | 79 (83) |  |  |  |  |
| Other, please specify | 75 (79) |  |  |  |  |
| Poor appetite | 74 (78) |  |  |  |  |
| Sleep disturbances | 70 (74) |  |  |  |  |
| Anxiety | 64 (67) |  |  |  |  |
| Depression | 52 (55) |  |  |  |  |
| Pain | 75 (7.9) |  |  |  |  |
| General coping | 53 (56) |  |  |  |  |
